# The Rate of Appropriate Adult Transthoracic Echocardiogram at King Abdulaziz University Hospital Based on Appropriate Use Criteria of 2011, 2017, and 2019

**DOI:** 10.7759/cureus.16262

**Published:** 2021-07-08

**Authors:** Afnan A Alotaibi, Mohammed Zahrani, Aseel Baflah, Aseel Alkhattabi, Amaal Algaydi, Farah Alsulami, Shehana Z Tayyeb

**Affiliations:** 1 Neurology, King Abdulaziz University Hospital, Jeddah, SAU; 2 Internal Medicine, King Abdulaziz University, Jeddah, SAU; 3 Cardiology, King Abdulaziz University Hospital, Jeddah, SAU

**Keywords:** rate, appropriate, tte, kauh, criteria, auc

## Abstract

Background: Transthoracic echocardiography (TTE) is a basic method for cardiovascular disease diagnosis and treatment. Studies done to assess the appropriate use of TTE in the Kingdom of Saudi Arabia (KSA) are scarce.

Objectives: To assess the pattern of ordering TTE in King Abdulaziz University Hospital (KAUH) and the appropriateness of its ordering.

Methods: A retrospective study was done from October to November 2018 at KAUH, Echo lab, Jeddah City, KSA. Patients, more than 18 years who had TTE at KAUH were included.

Results: The criteria used were the 2019 criteria for most patients and the orders were appropriate for 77.9% of the 954 patients. Orders were significantly inappropriate for patients who had older age, and the number of indications were significantly higher for those whose orders were - "maybe appropriate" (M). The anesthesia department for outpatients and the surgical department for inpatients ordered a significantly high number of inappropriate requests. Inpatients had a significantly higher percentage of "appropriate" (A) orders, and a significant positive correlation was present between patients’ age and number of indications.

Conclusion: There is a need to maximize compliance with AUCs and its effect on clinical results should be evaluated.

## Introduction

Over the last few decades, healthcare expenditures have increased dramatically in imaging throughout the world. The use of non-invasive imaging has increased faster than any other form of healthcare [1]. 

Transthoracic echocardiography (TTE) is a fundamental tool in the diagnosis and management of cardiovascular disease (CVD), which accounts for almost half of cardiac imaging services [2]. TTE is a widely available and flexible tool that has contributed to halving the frequency of major diagnostic errors in the last 20 years [3]. It provides precise diagnostic information regarding the physiology and anatomy of the cardiac chambers and allows high-quality visualization of cardiac valves, major vessels, and pericardium in a noninvasive and rapid manner [3-5].

In 2010, the investment of the United States (US) Healthcare was almost 2.1 trillion dollars and was expected to double in the following 30 years, which represents over 75% of costs in cardiology [6]. Echocardiography accounts for more than half of cardiovascular diagnostic imaging in the US; similar increases have been seen in Canada [7]. To respond to the dramatic increase in the use of diagnostic imaging, the healthcare community needs to recognize how to integrate this technology into daily clinical care [7].

The American College of Cardiology Foundation (ACCF) cooperates with the American Society of Echocardiography and other imaging subspecialty societies to design appropriate use criteria (AUC) for TTE [8]. AUC were assigned to echocardiography to direct physicians when ordering TTE, to improve patient care and health outcomes, and encourage the appropriate use of procedure [9]. Initially, the AUC were published in 2007 and later updated in 2011 to “respond to the need for the rational use of imaging services in the delivery of high-quality care” [10].

Nearly, all TTEs performed in a wide variety of clinical settings have been judged depending on the AUC [11,12]. These criteria categorize indications for echocardiography as appropriate, inappropriate, or uncertain [11,12].

The aim of applying the appropriateness criteria involves improving patient outcomes, including survival and health status, as well as decreasing the number of unessential imaging studies [11,13]. An “appropriate (score of 7 to 9),” study is deﬁned as an imaging study that is widely suitable as a reasonable approach for an indication, an “inappropriate (score of 1 to 3).” study is deﬁned as generally not suitable and not reasonable, and a study is considered as “uncertain (score of 4 to 6),” if it is suitable or reasonable, however, more research or patient information is needed [14,15]. 

Based on a careful literature review, no previous study regarding the appropriate use of TTE was conducted in King Abdulaziz University Hospital (KAUH), Kingdom of Saudi Arabia (KSA). The aim of this study was to provide insight into the pattern of ordering TTE in KAUH and the appropriateness of its ordering.

## Materials and methods

A retrospective study was done from October to November 2018 in KAUH, Echo lab, Jeddah city, KSA. The inclusion criteria were all patients who had TTE at KAUH and were >18 years of age between the study period and the exclusion criteria were patients <18 years and who were receiving dobutamine.

The data were collected from patients’ medical records, where an “appropriate score is 7 to 9” and an “inappropriate score is 1 to 3; the study is deﬁned as an imaging study that is widely suitable as a reasonable approach for an indication. The study is deﬁned as generally not suitable and not reasonable, and a study is considered as “uncertain (score of 4 to 6),” if it is suitable or reasonable; however, more research or patient information is needed [14,15]. The American College of Cardiology Foundation (ACCF) and the American Society of Echocardiography (ASE), established appropriate criteria (AUC) to promote more cost-effective utilization of echocardiography [16].

Ethical approval for the study was obtained from the research ethics committee of KAUH.

Statistical analysis

Data were analyzed using Statistical Package for the Social Sciences (SPSS) v. 25 (IBM Corp., Armonk, NY). Qualitative data were expressed as numbers and percentages. Chi-squared test (χ^2^) was applied to test the relationship between variables. Quantitative data were expressed as mean and standard deviation (mean ± SD), and the Kruskal Wallis test was applied for non-parametric variables. Correlation analysis using Spearman’s test was done, and a p-value of <0.05 was considered statistically significant.

## Results

Table 1 shows that the mean age of the studied 954 patients was 55.97 ± 16.63 years, and the mean number of indications was 41.6 ± 44.75. Of all the patients studied, 50.3% were female and 54.3% had a Saudi nationality. Most of the patients (62.5%) were inpatients and most of them (29.5%) were related to the department of medicine. Of the outpatients (37.5%), the majority (35.8%) were also related to the department of medicine.

**Table 1:** Distribution of studied patients according to their characters and whether they were in- or outpatient (no.= 954)

Variable	No. (%)
Age	55.97±16.63
Number of indications	41.6±44.75
Gender	
Female	480 (50.3)
Male	474 (49.7)
Nationality	
Non-Saudi	518(54.3)
Saudi	431 (45.2)
Unknown	5 (0.5)
In-or outpatient	
Inpatient	596 (62.5)
Outpatient	358 (37.5)
If outpatient, which surface?	
Family medicine	23(2.4)
Anesthesia	9 (0.9)
ENT	3 (0.3)
Medicine	281 (29.5)
Ob/Gyn	6 (0.6)
Ophthalmology	1 (0.1)
Orthopedic	1 (0.1)
Pediatric	1 (0.1)
Radiology	1 (0.1)
Surgery	32 (3.4)
If inpatient, what department?	
Anesthesia	2 (0.2)
CCU	40 (4.2)
ENT	8 (0.8)
ER	60 (6.3)
ICU	54 (5.7)
Medical	342 (35.8)
Ob/Gyn	9 (0.9)
Radiology	3 (0.3)
Surgical	78 (8.2)
Pre-operation result	
Not applicable	881 (92.3)
Negative - no	64 (6.7)
Positive - normal	9 (0.9)
Endocarditis	
Not applicable	947 (99.3)
Negative	7 (0.7)

**Figure 1:**
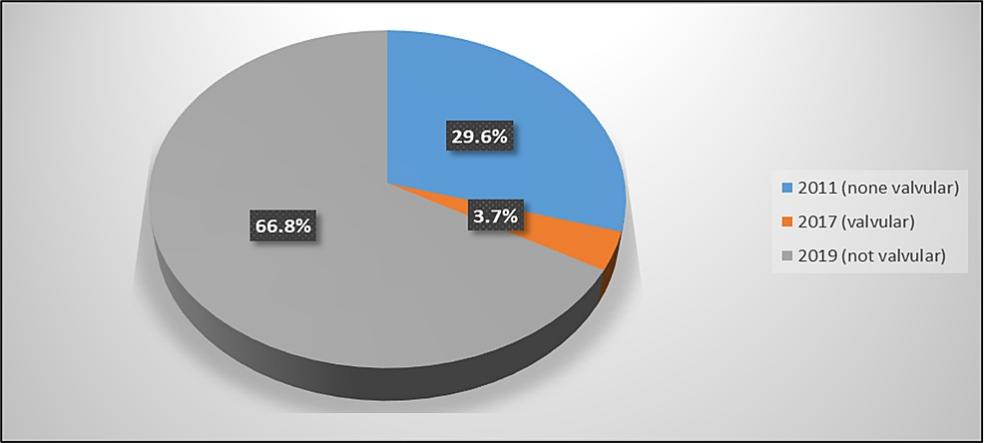
Percentage distribution of studied patients according to the used criteria.

**Figure 2:**
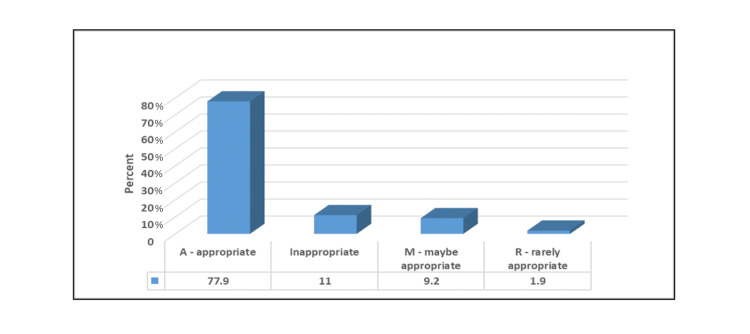
Percentage distribution of studied patients according to the appropriateness of the order.

Figure 1 illustrated that for most of the patients (66.8%), the used criteria were the 2019 (not valvular) criteria, and for a majority of them (77.9%), the order was appropriate (Figure 2).

Table 2 shows that orders were significantly inappropriate for patients who had an older age (60.85 ± 14.18 years), and the number of indications were significantly higher for those whose orders were maybe appropriate (M; p≤0.05). For patients whose data about (pre-operation result, and if there was a need to be reviewed) were not applicable, there was a significantly higher percentage of appropriate (A) orders (p≤0.05). Among outpatients, the departments of ophthalmology, orthopedic, pediatric, and radiology had a significantly higher percentage of appropriate requests when compared to other departments (p≤0.05). While among inpatients, the ENT department had a significantly higher percentage of appropriate requests when compared to other departments (p≤0.05). The anesthesia department for outpatients and surgical department for inpatients ordered a significantly higher percentage of inappropriate requests (p≤0.05). On the other hand, a non-significant relationship was found between the appropriateness of the order and patients’ gender, nationality, and presence of endocarditis (p≥0.05).

**Table 2:** Relationship between the appropriateness of the order and patients; characters and whether they were in- or outpatient (no.=954)

Variable	Appropriateness of the order							Test	p-Value
	A - appropriate		Inappropriate		M - may be appropriate		R - rarely appropriate		
Age	55.56 ± 16.56		60.85 ± 14.18		54.67 ± 18.36		50.83 ± 19.85	3*	0.015
Number of indications	38.81 ± 41.33		44.57 ± 54.68		59.34 ± 57.94		52.5 ± 9.55	3*	<0.001
Gender									
Female	369 (76.9)	58 (12.1)		45 (9.4)		8 (1.7)		1.41**	0.702
Male	374 (78.9)	47 (9.9)		43 (9.1)		10 (2.1)			
Nationality									
Non-Saudi	412 (79.5)	53 (10.2)		39 (7.5)		14 (2.7)		10.29**	0.113
Saudi	326 (75.6)	52 (12.1)		79 (11.4)		4 (0.9)			
Unknown	5 (100)	0 (0.0)		0 (0.0)		0 (0.0)			
Pre-operative result									
Not applicable	736 (83.5)	41 (4.7)		86 (9.8)		18 (2)		474.66**	<0.001
Negative-abno	6 (9.4)	56 (87.5)		2 (3.1)		0 (0.0)			
Positive-normal	1 (11.1)	8 (88.9)		0 (0.0)		0 (0.0)			
Endocarditis									
Not applicable	738 (77.9)	104 (11)		88 (9.3)		17 (1.8)		9.25**	0.026
Negative	5 (71.4)	1 (14.3)		0 (0.0)		1 (1.3)			
Need to be reviewed									
Not applicable	694 (79)	90 (10.2)		78 (8.9)		17 (1.9)		9.25**	0.026
Yes	49 (65.3)	15 (20)		10 (13.3)		1 (1.3)			
If outpatient, which surface?									
Family medicine	16 (69.6)	2 (6.7)		5 (21.7)		0 (0.0)		130**	<0.001
Anesthesia	3 (33.3)	4 (44.4)		2 (22.2)		0 (0.0)			
ENT	1 (33.3)	1 (33.3)		0 (0.0)		1 (33.3)			
Medicine	205 (73)	1 (7.5)		48 (17.1)		7 (2.5)			
Ob/Gyn	1 (16.7)	2 (33.3)		3 (50)		0 (0.0)			
Ophthalmology	1 (100)	0 (0.0)		0 (0.0)		0 (0.0)			
Orthopedic	1 (100)	0 (0.0)		0 (0.0)		0 (0.0)			
Pediatric	1 (100)	0 (0.0)		0 (0.0)		0 (0.0)			
Radiology	1 (100)	0 (0.0)		0 (0.0)		0 (0.0)			
Surgery	15 (46.9)	14 (43.8)		3 (9.4)		0 (0.0)			
If inpatient, what department?									
Anesthesia	1 (50)	1 (50)		0 (0.0)		0 (0.0)		197.06**	<0.001
CCU	39 (97.5)	0 (0.0)		1 (2.5)		0 (0.0)			
ENT	8 (100)	0 (0.0)		0 (0.0)		0 (0.0)			
ER	54 (90)	0 (0.0)		6 (10)		0 (0.0)			
ICU	45 (83.3)	4 (7.4)		4 (7.4)		1 (1.9)			
Medical	310 (90.6)	16 (4.7)		9 (2.6)		7 (2)			
Ob/Gyn	4 (44.4)	3 (33.3)		2 (22.2)		0 (0.0)			
Radiology	2 (66.7)	1 (33.3)		0 (0.0)		0 (0.0)			
Surgical	35 (44.9)	36 (46.2)		5 (6.4)		2 (2.6)			

**Figure 3:**
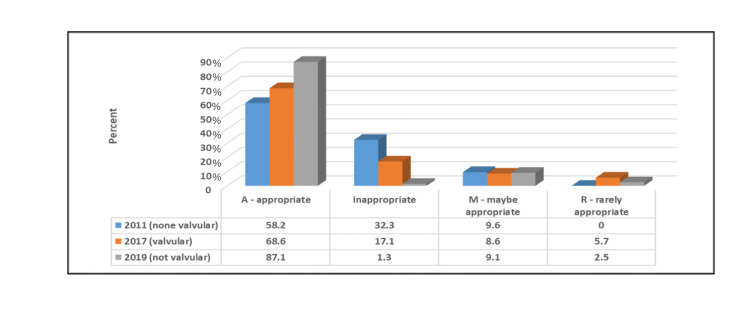
Relationship between the appropriateness of the order of the used criteria.

Figure 3 shows that patients for whom 2019 (not valvular) was the applied criteria, there was a significantly higher percentage of A orders (p≤0.05), and for patients for whom 2011 (non-valvular) was the applied criteria, there was a significantly higher percentage of rarely appropriate (R) orders (p≤0.05). 

**Figure 4:**
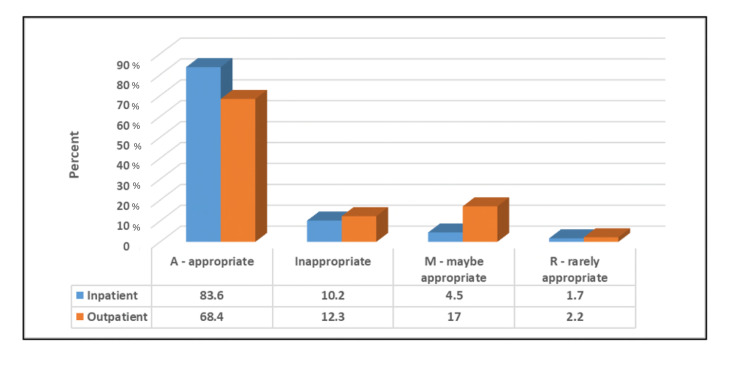
Relationship between the appropriateness of the order and whether patients were in- or outpatients.

Figure 4 shows that inpatients had a significantly higher percentage of A orders (p≤0.05). There is also a significant positive correlation between the patients’ age and the number of indications (r=0.19, p-value≤0.001).

Table 3 shows “symptoms or conditions potentially related to suspected cardiac etiology including but not limited to chest pain, shortness of breath, palpitations, TIA, stroke, or peripheral embolic event” A (9) was the commonest indication in AUC 2011, followed by “routine perioperative evaluation of ventricular function with no symptoms or signs of cardiovascular disease” I (2). In AUC 2017, the indications “initial postoperative evaluation of bioprosthetic or mechanical valve for the establishment of baseline (6w to 3m postoperative)” A (9) and “suspected IE (native valve, prosthetic valve, endocardial lead) and positive blood cultures or a new murmur” A (9) were the commonest. As for AUC 2019, the most used indication was “initial evaluation when symptoms or signs suggest heart disease” A (8) and then “initial evaluation of the patient to exclude cardiac origin of TIA or ischemic stroke - intracardiac masses (thrombus, vegetation) - valvular pathology” A (8) come after. Table 3 outlines these results for each criterion.

**Table 3:** The most ordered indications according to AUC 2011, AUC 2017 and AUC 2019 criteria with the frequencies and appropriateness of each indication

AUC used	Indications no.	Description	No. patients	Appropriateness
2019	9	Initial evaluation when symptoms or signs suggest heart disease	159	A (9)
	82	Initial evaluation of the patient to exclude cardiac origin of TIA or ischemic stroke - intracardiac masses (thrombus, vegetation) - valvular pathology	55	A (8)
	1	Initial cardiac evaluation of a known systemic, congenital, or acquired disease that could be associated with structural heart disease	34	A (8)
	68	Re-evaluation of known structural heart disease with change in clinical status or cardiac examination or to guide therapy (assume ischemic workup has been performed and remains valid)	33	A (8)
2017	57	Initial post-operative evaluation of bioprosthetic or mechanical valve for the establishment of baseline (6 weeks to 3 months postoperative)	4	A (9)
	16	Suspected IE (native valve, prosthetic valve, endocardial lead) and positive blood cultures or a new murmur	4	A (9)
	23	Symptomatic severe AS by calculated valve area (stage D2) and low flow/low gradient low LVEF	3	R (3)
	58	Reevaluation (<3 years after valve implantation) of bioprosthetic or mechanical valve or suspected valve dysfunction.	3	M (5)
2011	1	Symptoms or conditions potentially related to suspected cardiac etiology including but not limited to chest pain, shortness of breath, palpitations, TIA, stroke, or peripheral embolic event	64	A (9)
	13	Routine perioperative evaluation of ventricular function with no symptoms or signs of cardiovascular disease	64	I (2)
	169	Ischemic equivalent	16	A (8)
	161	Vascular surgery with stress echocardiography: ≥1 clinical risk factor poor or unknown functional capacity (<4 METs)	15	A (7)

## Discussion

AUC was established in response to the high use of non-invasive cardiac imaging services and the consequent healthcare cost [17]. AUC was meant to notify medical practitioners, patients, and health protocol constructors of how the use of this criteria would help enhance the symptoms of the patients, the outcome, and prevent over or under-applications [18].

In this retrospective study, we hand over identification of practice patterns and determination of appropriateness rates in a university hospital in KSA. To the best of our knowledge, this is the first study done to give an insight into the pattern of TTE ordering in KAUH and its appropriateness according to AUC.

In 2007, the first AUC study for adult TTE was published [19]. Studies evaluating the applicability of the AUC revealed that most TTE (87% to 89% of classifiable studies) were considered for appropriate (A) indication. However, many studies were unclassifiable, given a lack of matching AUC indications. Revisions were then incorporated into the 2011 AUC and allowed a marked decrease in the proportion of unclassifiable studies [20-22].

This study reported that 77.9% of orders were appropriate and 11% were inappropriate. This result concedes with what was revealed from a previous study done in Portugal, where 78.7% of classifiable echocardiograms were appropriate and 15.3% inappropriate [23]. At the same time, this result agrees with the results reported by other studies, in which the appropriateness rates range from 71.0% to 96.5% [24-27].

In this study, it was found that the highest percentage of requests for TTE were ordered for appropriate indication (77.9%). The most common “appropriate” indication for TTE was indication W “initial evaluation when symptoms or signs suggesting heart disease” and 11% were inappropriate (64 studies) with the most frequent “inappropriate” indication being indication 13 “routine perioperative evaluation of ventricular function with no symptoms or signs of cardiovascular disease.” Three studies had a “rarely appropriate” indication 23 “symptomatic severe AS by calculated valve area (stage D2) and low flow/low gradient low LVEF” and also three studies had a “maybe appropriate” indication 58 “reevaluation (<3 years after valve implantation) of bioprosthetic or mechanical valve or suspected valve dysfunction.” According to AUC for multimodality in valvular heart disease 2017 [17].

In the present study, in most patients (66.8%), the used criterion was the 2019 criteria, whereas, in 77.9% of them, the order was appropriate. This result is consistent with a previous retrospective study done in Morriston Hospital, Swansea, UK, which found that the proportions of appropriate and inappropriate indications were 86% and 11%, respectively [27]. Another retrospective study done in a university college in Cork, Ireland showed a result of 84.9% of appropriate orders and 10.9% of inappropriate orders [17]. The results revealed from the present study correlate with a systemic review that shows 84.7% of appropriate orders in the USA and 81.5% outside the USA [17].

Another cohort study was done at the University of Ottawa in Canada and it was found that the appropriate requests represented 67.9% and the inappropriate requests represented 10.4% based on the 2011 AUC criteria [28]. In a previous cross-sectional study done in the University Hospital Notre Dame des Secours, Byblos, Lebanon, 74.66% of the requests were appropriate and 16.96% were inappropriate [29].

The results of this study draw attention when compared to previous studies, the results of this work draw attention to the adherence to the AUC in different regions, and provides a chance of possible future protocols in the strict application of the AUC in our center and for further education to the trainees.

In this study, there was a variation of appropriateness in ordering TTE between inpatients and outpatients, where inpatients had a significantly higher percentage of A orders (83.6% and 68.4%, respectively) and 10.2% and 12.3% of inappropriate requests, respectively. A previous study found that inappropriate requests were more frequent in outpatients than in inpatients, a matter that was expected as inpatients usually present new symptoms or signs suggesting cardiac disease or worsening of known CVD, and both scenarios are rated as appropriate [23]. The same results were observed in other studies where a higher proportion of inappropriate exams were found among outpatients [18,24]. The previously mentioned retrospective study done in Cork, Ireland showed that outpatients had a significantly higher inappropriate referral rate compared to inpatients (13.8% vs. 7.1%) [15]. The results revealed from the present study and from previous studies indicated a higher inappropriate request rate in the outpatients’ group, which is an expected matter as outpatients are usually referred for routine TTE with no new symptoms or change in the clinical status that is mostly considered inappropriate.

In this study, most of the patients were inpatients and 29.5% were related to the department of medicine. A majority of the outpatients were also related to the department of medicine. Among outpatients, the ophthalmology, orthopedic, pediatric, and radiology departments had a significantly higher percentage of appropriate requests, and among inpatients, the ENT department had a significantly higher percentage of appropriate requests as compared to other departments. In the retrospective study done in Morriston hospital, Swansea, UK, the proportion of appropriate requests were highest (89%) for medical specialty and (80.8%) for surgical specialty [27]. This could be due to the high request of 2011 indication (routine preoperative evaluation of ventricular function with no symptoms or signs of CVD), which was inappropriate and was shown in both our study and the Swansea study.

In a previous study, most of the echocardiograms were requested by the cardiology department, followed by internal medicine, pneumology, cardiothoracic surgery, and oncology; and cardiologists were found to order inappropriate TTE more frequently than other specialties [23].

Limitations

The main limitation of this study was that the data were collected through a complete review of previous electronic medical records (EMR) and we did not have full information for patients included in the study. In addition, the rating of the clinical scenarios for appropriateness was a diﬃculty.

## Conclusions

In this study, all patients who had TTE at KAUH and were >18 years were included. For most patients, the used criterion was the 2019 criteria, and for 77.9%, the order was appropriate. Orders were significantly inappropriate for patients who had older age, and the number of indications were significantly higher for those whose orders were M. Anesthesia department for outpatients and surgical department for inpatients ordered a significantly higher percentage of inappropriate requests. Patients for whom the 2019 criteria were applied had a significantly higher percentage of A orders. Inpatients had a significantly higher percentage of A orders and a significant positive correlation was present between patients’ age and a number of indications. There is a need for strategies to maximize compliance with AUCs and their effect on clinical results should be evaluated through future studies.

## References

[1] Institute of Medicine (US) Committee on Technological Innovation in Medicine; Gelijns AC, Halm EA (1991). The Changing Economics of Medical Technology. Economics of Medical Technology. Washington (DC): National Academies Press (US.

[2] Steeds RP (2011). Echocardiography: frontier imaging in cardiology. Br J Radiol.

[3] Price S, Via G, Sloth E, Guarracino F, Breitkreutz R, Catena E, Talmor D (2008). Echocardiography practice, training and accreditation in the intensive care: document for the World Interactive Network Focused on Critical Ultrasound (WINFOCUS). Cardiovasc Ultrasound.

[4] Omerovic S, Jain A (2020). Echocardiogram. https://www.ncbi.nlm.nih.gov/books/NBK558940/.

[5] Committee on Diagnostic Error in Health Care; Board on Health Care Services; Institute of Medicine; The National Academies of Sciences, Engineering Engineering, and Medicine (2015). Improving Diagnosis in Health Care. https://pubmed.ncbi.nlm.nih.gov/26803862/.

[6] Branning G, Vater M (2016). Healthcare spending: plenty of blame to go around. Am Health Drug Benefits.

[7] Institute of Medicine (US) Committee on Social Security Cardiovascular Disability Criteria (2010). Cardiovascular Disability: Updating the Social Security Listings. https://pubmed.ncbi.nlm.nih.gov/24983036/.

[8] Douglas PS, Garcia MJ, Haines DE (2011). ACCF/ASE/AHA/ASNC/HFSA/HRS/SCAI/SCCM/SCCT/SCMR 2011 Appropriate Use Criteria for Echocardiography. A report of the American college of cardiology foundation appropriate use criteria task force, American society of echocardiography, American heart association, American society of nuclear cardiology, heart failure society of America, heart rhythm society, society for cardiovascular angiography and interventions, society of critical care medicine, society of cardiovascular computed tomography, society for cardiovascular magnetic resonance American College of Chest Physicians. J Am Coll Cardiol.

[9] Bhatia RS, Ivers NM, Yin XC (2017). Improving the Appropriate Use of Transthoracic Echocardiography: The Echo WISELY Trial. J Am Coll Cardiol.

[10] Welch HG, Hayes KJ, Frost C (2012). Repeat testing among medicare beneficiaries. Arch Int Med.

[11] Hackett I, Ward RP (2020). Appropriate use criteria for echocardiography in the era of value-based care: mission accomplished or future mandates?. Curr Cardiol Rep.

[12] Willens HJ, Nelson K, Hendel RC (2013). Appropriate use criteria for stress echocardiography: impact of updated criteria on appropriateness ratings, correlation with pre-authorization guidelines, and effect of temporal trends and an educational initiative on utilization. JACC Cardiovasc Imaging.

[13] Kirkpatrick JN, Ky B, Rahmouni HW (2009). Application of appropriateness criteria in outpatient transthoracic echocardiography. J Am Soc Echocardiogr.

[14] Matulevicius SA, Rohatgi A, Das SR, Price AL, DeLuna A, Reimold SC (2013). Appropriate use and clinical impact of transthoracic echocardiography. JAMA Intern Med.

[15] Kerley RN, O'Flynn S (2019). A systematic review of Appropriate Use Criteria for transthoracic echocardiography: are they relevant outside the United States?. Ir J Med Sci.

[16] Douglas PS, Garcia MJ, Haines DE (2011). ACCF/ASE/AHA/ASNC/HFSA/HRS/SCAI/SCCM/SCCT/SCMR 2011 appropriate use criteria for echocardiography. A report of the American College of Cardiology Foundation Appropriate Use Criteria Task Force, American Society of Echocardiography, American Heart Association, American Society of Nuclear Cardiology, Heart Failure Society of America, Heart Rhythm Society, Society for Cardiovascular Angiography and Interventions, Society of Critical Care Medicine, Society of Cardiovascular Computed Tomography, and Society for Cardiovascular Magnetic Resonance Endorsed by the American College of Chest Physicians. J Am Coll Cardiol.

[17] Singh A, Ward RP (2016). Appropriate Use Criteria for Echocardiography: Evolving Applications in the Era of Value-Based Healthcare. Curr Cardiol Rep.

[18] Patil HR, Coggins TR, Kusnetzky LL, Main ML (2012). Evaluation of appropriate use of transthoracic echocardiography in 1,820 consecutive patients using the 2011 revised appropriate use criteria for echocardiography. Am J Cardiol.

[19] Martin NM, Picard MH (2009). Use and appropriateness of transthoracic echocardiography in an academic medical center: a pilot observational study. J Am Soc Echocardiogr.

[20] Douglas PS, Khandheria B, Stainback RF (2007). ACCF/ASE/ACEP/ASNC/SCAI/SCCT/SCMR 2007 appropriateness criteria for transthoracic and transesophageal echocardiography: a report of the American College of Cardiology Foundation Quality Strategic Directions Committee Appropriateness Criteria Working Group, American Society of Echocardiography, American College of Emergency Physicians, American Society of Nuclear Cardiology, Society for Cardiovascular Angiography and Interventions, Society of Cardiovascular Computed Tomography, and the Society for Cardiovascular Magnetic Resonance endorsed by the American College of Chest Physicians and the Society of Critical Care Medicine. J Am Coll Cardiol.

[21] Martin NM, Picard MH (2009). Two years of appropriateness criteria for echocardiography: what have we learned and what else do we need to do?. J Am Soc Echocardiogr.

[22] Ward RP, Mansour IN, Lemieux N, Gera N, Mehta R, Lang RM (2008). Prospective evaluation of the clinical application of the American College of Cardiology Foundation/American Society of Echocardiography Appropriateness Criteria for transthoracic echocardiography. JACC Cardiovasc Imaging.

[23] Fonseca P, Sampaio F, Ribeiro J, Gonçalves H, Gama V (2015). Appropriate use criteria for transthoracic echocardiography at a tertiary care center. Rev Port Cardiol.

[24] Bhatia RS, Carne D, Picard M (2012). Comparison of the 2007 and 2011 appropriate use criteria for transthoracic echocardiography in various clinical settings. J Am Soc Echocardiogr.

[25] Bailey S, Mosteanu I, Tietjen P (2012). The use of transthoracic echocardiography and adherence to appropriate use criteria at a regional hospital. J Am Soc Echocardiogr.

[26] Ballo P, Bandini F, Capecchi I (2012). Application of 2011 ACC/ASE appropriateness of use criteria in hospitalized patients referred for transthoracic echocardiography in a community setting. J Am Soc Echocardiogr.

[27] Gurzun MM, Ionescu A (2014). Appropriateness of use criteria for transthoracic echocardiography: are they relevant outside the USA?. Eur Heart J Cardiovasc Imaging.

[28] Chan KL, Liu X, Ascah KJ, Beauchesne LM, Burwash IG (2004). Comparison of real-time 3-dimensional echocardiography with conventional 2-dimensional echocardiography in the assessment of structural heart disease. J Am Soc Echocardiogr.

[29] Rameh V, Kossaify A (2016). Appropriate use criteria in echocardiography: an observational institutional study with the perspective of a quality improvement project. Clin Med Insights Cardiol.

